# A Mouse-Adapted Model of HCoV-OC43 and Its Usage to the Evaluation of Antiviral Drugs

**DOI:** 10.3389/fmicb.2022.845269

**Published:** 2022-05-17

**Authors:** Peifang Xie, Yue Fang, Zulqarnain Baloch, Huanhuan Yu, Zeyuan Zhao, Rongqiao Li, Tongtong Zhang, Runfeng Li, Jincun Zhao, Zifeng Yang, Shuwei Dong, Xueshan Xia

**Affiliations:** ^1^The Affiliated AnNing First Hospital, Faculty of Life Science and Technology, Kunming University of Science and Technology, Kunming, China; ^2^State Key Laboratory of Respiratory Disease, National Clinical Research Center for Respiratory Disease, Guangzhou Institute of Respiratory Health, The First Affiliated Hospital of Guangzhou Medical University, Guangzhou, China

**Keywords:** human coronavirus OC43, adaptation, mouse model, respiratory disease, model application

## Abstract

The human coronavirus OC43 (HCoV-OC43) is one of the most common causes of common cold but can lead to fatal pneumonia in children and elderly. However, the available animal models of HCoV-OC43 did not show respiratory symptoms that are insufficient to assist in screening antiviral agents for respiratory diseases. In this study, we adapted the HCoV-OC43 VR-1558 strain by serial passage in suckling C57BL/6 mice and the resulting mouse-adapted virus at passage 9 (P9) contained 8 coding mutations in polyprotein 1ab, spike (S) protein, and nucleocapsid (N) protein. Pups infected with the P9 virus significantly lost body weight and died within 5 dpi. In cerebral and pulmonary tissues, the P9 virus replication induced the production of G-CSF, IFN-γ, IL-6, CXCL1, MCP-1, MIP-1α, RANTES, IP-10, MIP-1β, and TNF-α, as well as pathological alterations including reduction of neuronal cells and typical symptoms of viral pneumonia. We found that the treatment of arbidol hydrochloride (ARB) or Qingwenjiere Mixture (QJM) efficiently improved the symptoms and decreased *n* gene expression, inflammatory response, and pathological changes. Furthermore, treating with QJM or ARB raised the P9-infected mice’s survival rate within a 15 day observation period. These findings suggested that the new mouse-adapted HCoV-OC43 model is applicable and reproducible for antiviral studies of HCoV-OC43.

## Introduction

Coronaviruses (CoVs) are positive-strand RNA viruses with a genome of 30 kb in length, prone to mutation, and recombination, leading to frequent viral evolution and infecting a wide range of hosts (Lai, 1992; Woo et al., 2010). They belong to the subfamily *Orthocoronavirinae* which has been divided into four genera (*alpha, beta, delta, and gammacoronavirus*). Seven strains of CoVs known to be susceptible to humans (namely, as HCoVs) are limited to the *alpha* (HCoV-229E and HCoV-NL63) and *beta* (HCoV-OC43, HCoV-HKU1, SARS-CoV, MERS, and SARS-CoV-2) genera (Malik, 2020). Among them, HCoV-OC43 is one of the most common causes of common colds in the general population. Like other CoVs, the first two-thirds of the genome of HCoV-OC43 contain open reading frames (ORFs) 1a and 1b, which are translated by ribosomal frameshifting to generate poly protein pp1ab, and the pp1ab is processed into non-structural proteins (NSP1-16) to form the replicasetranscriptase complex (RTC). Downstream of ORF1b, there are ORFs encoding structural and accessory proteins (Vijgen et al., 2005; Fehr and Perlman, 2015). The Spike (S) protein, one of the most important structural proteins of HCoV-OC43, is involved in virus attachment and entry processes and plays an important role in virus pathogenicity and host tropism (Peng et al., 2012; Song et al., 2020). Mutations in S protein were thought to be the primary reason for cross-species transmission and virus evolution (Peng et al., 2012).Besides, the hemagglutinin-esterase (He) specifically acts on 9-O-acetylated sialic acids to remove acetyl groups, aid in receptor binding and viral release, and promote efficient viral replication by balancing viral attachment and release (Lang et al., 2020). The nucleocapsid (N) protein, another significant structural protein, encapsulates the viral RNA and, along with NSPs, plays a crucial role in virus replication, transcriptional processes, and genome assembly (Abdel-Moneim et al., 2021).The frequent virus evolution has an impact on the adaptation of viruses to specific hosts (Coleman and Frieman, 2014).

Apart from mild upper respiratory tract symptoms, HCoV-OC43 infection in infants, the elderly, or immune-compromised adults would cause fatal encephalitis or severe lower respiratory tract illness, including bronchiolitis, asthma, and pneumonia (Vabret et al., 2003; Morfopoulou et al., 2016). It has been reported that the primary pathological mechanism of HCoV infection is the interaction between the virus and the host, which would lead to a severe immune response (de Wilde et al., 2018). The occurrence of a storm of pro-inflammatory cytokines results in tissue damage (Vabret et al., 2006; de Wilde et al., 2018). Moreover, endemic infection of HCoVs (OC43, HKU1, NL63, and 229E) caused fatalities in healthy adults and HCoV-OC43 (hazard ratio, 2.50) was substantially linked with coronavirus death (Kim et al., 2021; Veiga et al., 2021). Although there have not yet been a significant number of severe cases worldwide, the importance of including HCoVs in diagnostic panels used by official surveillance systems and the necessity of more cautious treatment for coronavirus patients has been increasingly recognized by researchers (Kim et al., 2021; Veiga et al., 2021). These studies suggest that anti-inflammatory and antiviral research are both necessary for the development of antiviral therapeutics for HCoV infections.

There are no specific antiviral therapies available for HCoV-OC43 and other HCoVs (Rajapakse and Dixit, 2021). Qingwenjiere Mixture (QJM) is a Traditional Chinese Medicine (TCM) compound with clinical efficacy against SARS-CoV-2, and we previously proved that it was effective against *in vitro* infection of SARS-CoV-2, HCoV-OC43, HCoV-229E, and HCoV-NL63 (Xie et al., 2021). Arbidol hydrochloride (ARB), a well-known broad-spectrum antiviral compound, was widely used in clinical anti-respiratory virus therapy (Blaising et al., 2014). It is effective against COVID-19 in clinical practice as well as being demonstrated to have an antiviral effect against the HCoV-OC43 virus *in vitro* (Nojomi et al., 2020; Leneva et al., 2021). However, the efficacy of QJM and ARB against *in vivo* HCoV-OC43 infection is unconfirmed. Animal models are useful for studying viral pathogenesis and evaluating antiviral and vaccine candidates. A few animal models for HCoV-OC43 infection have been developed, but it is unknown whether the infection causes lung inflammation or respiratory illness in these animals and hinders the screening of antiviral medicines (Butler et al., 2006; Keyaerts et al., 2009). Here, we made a new mouse-adapted HCoV-OC43 model with typical pulmonary disease symptoms and looked into how it could be used in pathogenesis studies and drug testing.

## Materials and Methods

### Ethical Statement

Animal experiments were carried out in accordance with the Chinese Laboratory Animal Regulations (Ministry of Science and Technology of the People’s Republic of China) and the National Laboratory Animal Standardization Technical Committee. The Animal Experiment Committee at Kunming University of Science and Technology in China approved this study, and the approval number is PZWH (Dian) K2020-0013.

### Viruses, Animals, and Drugs

The wild type of HCoV-OC43 VR-1558 was provided by Prof. Jincun Zhao (Guangzhou Medical University) and propagated in HRT-18 cells (ATCC CCL-244). The titer of viral stock was determined using the Reed–Muench method with a 50% tissue culture infective dose (TCID_50_) based on cytopathic effect of HRT-18 cells. Specified pathogen-free (SPF) C57BL/6 mice were purchased from the Experimental Animal Center of Kunming Medical University. Suckling mice weighing 4–6 g within 10 days of birth with no distinction between males and females were used for subsequent viral inoculation and *in vivo* evaluation of drug efficacy. Arbidol hydrochloride tablets (ARB, 0.1 g per tablet) were purchased from CSPC Ouyi Pharmaceutical Co., Ltd. Qinwenjiere Mixture (QJM) was provided by Chinese medicine hospitals in Yunnan Province with an original concentration of 600 mg/ml. These drugs were dissolved in pure water and diluted to specific concentrations before usage.

### Serial Passage of HCoV-OC43 in Suckling C57BL/6 Mice

**Figure 1:**
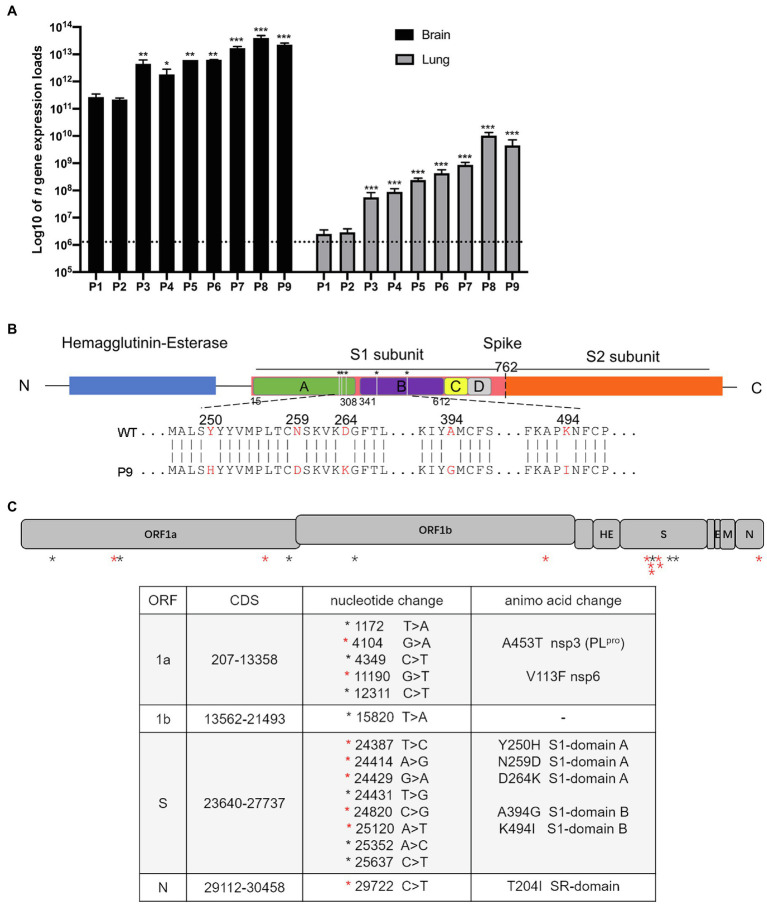
The expression of viral *n* gene in the brain and lungs of a series generations of mice and mutation sites between wild and adapted strains. **(A)** The expression of viral *n* gene in the brain and lung was detected at 4 dpi (*n* = 3–5). The data are presented as the log10 of the expression of viral *n* gene (copies/g), the mean and the standard error of the mean (SEM). ^*^p < 0.05; ^**^p < 0.01; and ^***^p < 0.001; vs. P1. **(B)** A schematic representation of the HCoV-OC43-1558 HE and S proteins (drawn to scale) and the five amino acid substitutions. The hemagglutinin-esterase protein is colored in blue, the S1 subunit and S2 subunit of S protein are colored in pink and orange, respectively, and the S1 domains A, B, C, and D are colored in green, purple, yellow, and gray, respectively. The substitution of Y250H, N259D, and D264K are located in domain A of the S1 subunit, while A394G and K494I are located in domain B. **(C)** Schematic diagram of HCoV-OC43 genome indicating mutations found in P9 virus. (Top) The 30,746 nucleotide RNA genome of HCoV-OC43 is shown in this to scale drawing with ORFs indicated by gray boxes. Black asterisks indicate nucleotide mutations which did not result in coding changes. Red asterisks indicate nucleotide mutations resulting in coding changes. (Bottom) The 15 nucleotide mutations resulted in 8 coding changes in ORF1a, S, and N, respectively.

Suckling mice, 3–5 mice per group, were intracerebrally injected with 25 μl of the wild type of HCoV-OC43 VR-1558 virus (100TCID_50,_ equals to 2.5 × 10^7^ copies), and the normal control group mice received the same volume of sterile PBS. Animals were sacrificed at 1, 2, 3, 4, and 5 days post-infection (dpi). Lung and brain tissues were collected and homogenized in sterile PBS with a weight to volume ratio of 1:10 and clarified by centrifugation. Viral RNA was extracted from 140 μl of the supernatant with the E.Z.N.A. Viral RNA Extraction 162 R6874-02 (Omega Bio-Tek, United Kingdom) kit. The expression of OC43 nucleocapsid (*n*) genes was determined by quantitative real-time RT-PCR (qRT-PCR) as described below, followed by selecting one brain tissue filtrate that had a higher viral load at 4 dpi for further intracerebral inoculation, in which the final copy number of the inoculated virus was 10^6^ copies/μl (data not shown). Each generation of the OC43 strain was used for gene sequencing. The clarified supernatant was stored at −80°C. Partial results of this part are shown in Figure 1 and Supplementary Table 2.

### Sequence Analysis

For sequence analysis of *s* and *Hemagglutinin-esterase* (*He*) genes, viral RNA from brain samples infected with WT to P9 viruses was reverse transcribed to cDNA by using the PrimeScript™ RT Master Mix Kit (Takara Bio, Japan). For genome sequence analysis, viral RNA from brain samples infected with WT and P9 viruses was transcribed *via* aforementioned method. Viral genes were amplified with the high-fidelity Taq enzyme (Vazyme) with corresponding primers (Supplementary Table 1). The sequences in 5′ and 3′ untranslated regions (UTR) were amplified with the SMARTer RACE 5′/3’ Kit (Takara Bio, Japan) according to the instruction. The purified PCR products with expected fragment sizes were sequenced by Tsingke Biological Technology Co., Ltd. Sequences were analyzed and assembled with SeqMan software (DNAStar Inc., Madison, WI, United States) and Mega software (Mega RAID SAS9240-8i, United States).

### Determination of the Median Lethal Dose of Suckling Mice (SMLD_50_) for P9 and WT Viruses

For the P9 virus, suckling mice were randomly divided into 7 groups, including 10^−2^, 10^−3^, 10^−4^, 10^−5^, 10^−6^, and 10^−7^ and normal control group, with 10 suckling mice per group. P9 stock was successively diluted into 10^−2^, 10^−3^, 10^−4^, 10^−5^, 10^−6^, and 10^−7^ in serial log10 dilutions. In the corresponding groups, each dilution was intracerebrally inoculated with mice for 25 μl per mouse. Animals in the normal control group received the same volume of sterile PBS. The mortality rate was recorded daily for 15 consecutive days, and the SMLD50 of P9 was calculated using the Reed-Muench method. Virus titers were expressed as the reciprocal of the virus suspension’s highest dilution at which 50% of inoculated suckling mice died (SMLD_50_; Reed and Muench, 1938). The SMLD_50_ of WT was determined by a similar procedure.

### Grouping and Modeling of Mice

Suckling mice were randomly divided into three groups: the normal control group, the wild strain group (WT), and the adapted strain group (P9) to compare the characteristics of WT and P9 viruses. Each mouse in the WT and P9 groups received 1000SMLD_50_ of WT or P9 virus intracerebrally. Animals in normal control group were injected with 25 μl sterile PBS per mouse. Experiments were conducted as follows:

a. Mortality and morbidity study (10 mice per group): The symptoms, body weight, and survival time were monitored regularly for 15 days. Individuals who died within 24 h of infection were categorized as abnormal deaths, and the mean survival time (days) of each group was calculated (Smee et al., 2010).

b. The proliferation kinetics of the P9 virus in brain and lung samples (21 mice per group): After fasting for 2 h, the suckling mice were inoculated. At 12, 24, 36, 48, 72, 96, and 120 hpi, the mice were sacrificed and the copy number of OC43 *n* gene in brain and lung tissues was detected by qRT-PCR.

c. Examination of the organ index changes and OC43 *n* gene copy number at 4 dpi (4 mice per group): suckling mice were anaesthetized and sacrificed at 4 dpi to dissect the brain, heart, liver, spleen, lung, and kidney. The organ tissues and the mouse body were weighed to calculate the organ indexes. The organ tissues were, respectively, homogenized with sterile PBS for qRT-PCR detection of the copy number of OC43 *n* gene (CT value of >35 was considered negative).

d. Changes in organ index and the copy number OC43 *n* gene expression at 5 dpi (P9) and 8 dpi (WT): At 5 dpi, three mice in the normal control group and four mice in the P9 group were sacrificed, while the other three normal control mice and three WT-infected suckling mice were sacrificed at 8 dpi. The brain and lung indices and the OC43 *n* gene expression were detected.

e. OC43 N protein expression in organs and the histopathological changes of suckling mice (3–5 mice per group, but nine mice per control group): At 3 and 4 dpi, the whole tissues from the P9 group were fixed with 4% formalin for histopathological and immunohistochemical detection. The control and WT groups were analyzed by a similar procedure at 3, 4, and 8 dpi.

f. The detection of the production of inflammatory cytokines and chemokines: For the WT group, the brain and lung tissues were harvested at 0.5, 4, and 8 dpi, while the tissues of P9-infected mice were collected at 0.5 and 4 dpi. The homogenized brain and lung samples were used to detect pro-inflammatory factors produced by the Bio-plex assay.

g. The detection of P9 infection rate: suckling mice were divided into normal control group and P9 group. 80 mice were individually challenged with 1000SMLD_50_ of P9 virus, while 10 mice in the control group received the same volume of sterile PBS. At 4 dpi, the entire brains and lungs from these two groups were isolated and homogenized with sterile PBS for qRT-PCR detection of the expression of OC43 *n* gene.

h. Evaluation of *in vivo* efficacy of ARB and QJM against P9 infection: the suckling mice were divided into 6 groups, including the normal control group, the P9 group, and four drug intervention groups, namely, ARB 25 mg·kg^−1^·d^−1^ and QJM high-, medium-, and low-dose groups (QJM 600 mg·kg^−1^·d^−1^, 300 mg·kg^−1^·d^−1^, and 150 mg·kg^−1^·d^−1^). Mice in P9 and drug intervention groups were individually challenged with 1000SMLD_50_ of P9 virus, and the normal control group animals received the same volume of sterile PBS. At 2 hpi, P9-infected mice were, respectively, given 25 mg·kg^−1^·d^−1^ of ARB and 600, 300, 150 mg·kg^−1^·d^−1^ of QJM *via* oral administration with 100 μl/mouse/day for 4 days. The examination was conducted as follows:

1. The effects of drugs on morbidity and mortality (10 mice per group): After the above-mentioned intervention procedure, the symptoms, body weight, survival time, and survival number of mice were recorded on a daily basis for 15 consecutive days. The survival curve was calculated (Smee et al., 2010).

2. At 4 dpi, the animals (10 mice per group) were sacrificed to isolate their entire brains and lungs. The organ indexes, the copy number of viral *n* gene, inflammation responses, and pathological changes of infected pups were analyzed as mentioned below.

### Organ Index Calculation and Quantitative Real-Time RT-PCR (qRT-PCR) Detection of the Expression of Viral *N* Gene

The organ indices were calculated as a ratio of organ weight (g) to body weight (g) multiplied by 100%. Viral RNA was extracted from the tissue samples as mentioned above, and 2 μl of viral RNA was reverse transcribed and amplified using the One Step PrimeScript™ RT-PCR Kit (Takara Bio, Japan). The amplification was carried out using an Applied Biosystems 7,500 Real-Time PCR System. Data were recorded by the 7,500 Real-Time PCR software and expressed as a function of Threshold Cycle (CT). The *n* gene of HCoV-OC43 was cloned into pEASY-T1 cloning vector 165 (Supplementary Table 1, Trans Gen Biotech, China) and the plasmid was used to generate the standard curve to calculate the copy number of the OC43 *n* gene (Chan et al., 2020). The OC43 *n* gene expression was calculated as copies/g = 10^^[(41.248-Ct)/3.281]^ × 0.429 × volume/organ weight (g), with a CT value greater than 35 considered negative.

### Detection of the Production of Inflammatory Cytokines/Chemokines by Bio-Plex Assay

Brain and lung tissues were homogenized as mentioned above and stored at −80°C before further analysis. Prior to detection, the samples were thawed on ice, clarified by centrifugation at 4°C for 10 min at 10,000 rpm/min, and the supernatant was collected. The protein concentration in the supernatants was measured using the Pierce BCA Protein Assay kit [Thermo Fisher Scientific (China) Co., Ltd.] according to the manufacturer’s protocol. All samples were adjusted to the same concentration. The concentration of cytokines and chemokines in the supernatants of tissue homogenates was measured by the Bio-Plex Pro-Mouse Cytokine assay using the Bio-Plex 200 Multiplex Testing System (Bio-Rad, United States) according to the manufacturer’s protocol. The data were analyzed using Bio-Plex Manager software (version 5.0; Bio-Rad, Labs).

### Histopathology and Immunohistochemistry

The whole brain and lung samples from each group were dissected at the indicated times and fixed with 4% paraformaldehyde for 24 h, then dehydrated with ethanol, permeated with xylene, embedded with paraffin, and sectioned into 4 ~ 6 μm slides. For the histopathological assay, slides were stained with hematoxylin and eosin (HE; Wuhan Google Biotechnology Co., Ltd., G1005). For immunohistochemical staining, slides were deparaffinized, rehydrated, and boiled in a citric acid (pH 6.0) antigen retrieval solution (ServiceBio, G1202). Endogenous peroxidase activity was blocked by 3% hydrogen peroxide at room temperature for 25 min. Slides were blocked with 3% BSA at room temperature for 30 min and incubated with antibody against the N protein of HCoV-OC43 (1:300, Millipore, MAB9012) overnight at 4°C. Sections were washed three times with PBS before being incubated for 50 min at room temperature with goat anti-mouse IgG (1,200, ServiceBio, GB23301) labeled with HRP. Then, the sections were stained with 3,3′-diaminobenzidine (DAB; ServiceBio, G1211) and counterstained with hematoxylin (ServiceBio, G1004, G1309, and G1340). Images were captured using a DMI3000B Manual Inverted Microscope (Leica) and analyzed with NIS-Elements F 4.00.00 software (Leica). The staining intensity and rate of positive cells were analyzed by the software AIpathwell (Servicebio). The Histochemistry score (H-Score) was used for semi-quantitative analysis of the OC43 N protein expression level, and the H-Score was calculated as ∑(pi×i), with pi representing the ratio of positive signal pixel area to cell number and i representing staining intensity scores (no staining = 0, weak staining = 1, moderate staining = 2, and strong staining = 3). The H-Scores ranged from 0 to 300, and larger number indicated higher staining intensity. Under double-blind conditions, HE score was recorded to evaluate pathological changes in brain and lung tissues. The HE scores of brain samples were recorded as follows: 0 points, no inflammatory cell infiltration and neuronal degeneration; 1 point, 25% inflammatory cell infiltration and neuronal degeneration; 2 points, 50% inflammatory cell infiltration and neuronal cells degeneration or decrease; and 75% ~ 100% inflammatory cell infiltration and neuronal cells degeneration or decrease. The HE scores of lung samples were described by Buchweitz et al. (2007).

### Statistical Analysis

Data analysis was performed using IBM SPSS Statistics 21.0 software. The survival curve was described by the Kaplan–Meier method and statistically analyzed by the log-rank test. One-way ANOVA was used for statistical analysis of the mean survival time, organ index, the expression of viral *n* gene, and the production of inflammatory factors. The Kruskal-Wallis test was used for the analysis of H-Scores and HE scores. *p* < 0.05 indicated a significant difference, and *p* < 0.01 and *p* < 0.001 indicated the difference was very significant.

## Results

### Acquisition of an Adapted HCoV-OC43 Strain With 8 Amino Acid Mutations in the Genome

To create an HCoV-OC43 mouse adopted strain, suckling C57BL/6 mice were intracerebrally inoculated with 100 TCID_50_ of wild-type virus (WT) by serial passage. Brain and lung tissue samples were collected from infected animals at 1 to 5 dpi, respectively, and brain samples with higher *n* gene expression at 4 dpi were used for serial inoculation. The expression of viral *n* gene in the brain and lung, respectively, approached 3.96 × 10^13^ copies/g and 1.03 × 10^11^ copies/g, at passage 8 (P8; Figure 1A). The sequences of the He and S regions from the nine generations of viruses (WT to P9) were first determined and analyzed. There were no mutations within *He* gene from WT to P9, but nine nucleic acid mutations were detected within ORF S. Among them, only six mutations resulted in five amino acid changes. Three changes (Y250H, N259D, and D264K) in the A domain of the S1 subunit and two mutations (A394G and K494I) in the B domain were detected (Figure 1B, Supplementary Table 2). In order to identify mutations associated with this adaptation, the genome sequences of WT and P9 were further investigated. Six nucleic acid mutations resulted in two amino acid changes, A453T (NSP3) and V113F (NSP6), in ORF1a (Figure 1C). One nucleic acid mutation leading to a T204I change was detected in ORF N (Figure 1C). No changes were identified in regions of 1b, ns2, ns12.9, E, and M (Figure 1C).

**The adapted strain (P9) had increased virulence against suckling C57BL/6 mice.**

**Figure 2:**
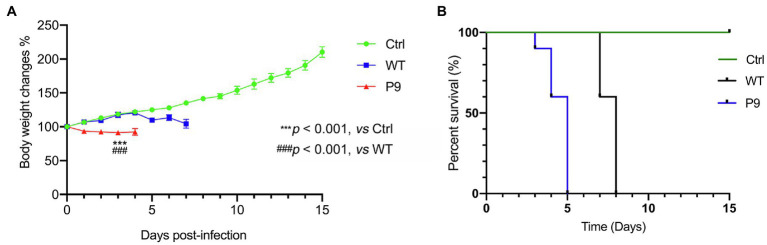
Body weight changes and survival rates in suckling mice infected with wild and adapted strains. After inoculation with a wild-type strain virus (WT) or an adapted-strain virus (P9), body weight changes **(A)**, and survival rates **(B)** of suckling mice within 15 days. The data are presented as the mean and SEM (*n* = 10).

The SMLD_50_ of P9 and WT in suckling mice was determined, which were 10^–5.625^/25 μl and 10^–3.625^/25 μl, respectively. The disease symptoms of infected animals were recorded regularly. At 1 dpi, the mice infected with the P9 virus began to lose weight. They were reluctant to move and ingest milk at 2 dpi, with unkempt hair and poor skin, and later developed a swaggering walk and a curling tendency. The mortality rate of these mice was 100% at 5 dpi (Figure 2B). Body weight gain and activity of WT group animals were normal (Figure 2A), but later they developed disorganized hair, a swaggering stride, or curled up at 7 and 8 dpi, and then died. In the normal control group, no abnormality was found in body weight gain or regular activity (Figure 2A). The mean survival time in group P9 was significantly shorter than in the WT group (4.5 ± 0.224 vs. 7.6 ± 0.163, *p* < 0.001; Figure 2B, Supplementary Table 3). These findings indicate that both the WT and the adapted P9 viruses were fatal to suckling mice, but P9 had an earlier median lethal time, a shorter average survival period, and a lower body weight under the same infective dose.

### The Adapted Strain (P9) Was Efficiently Replicated in Brain and Lung Samples of Suckling Mice

**Figure 3:**
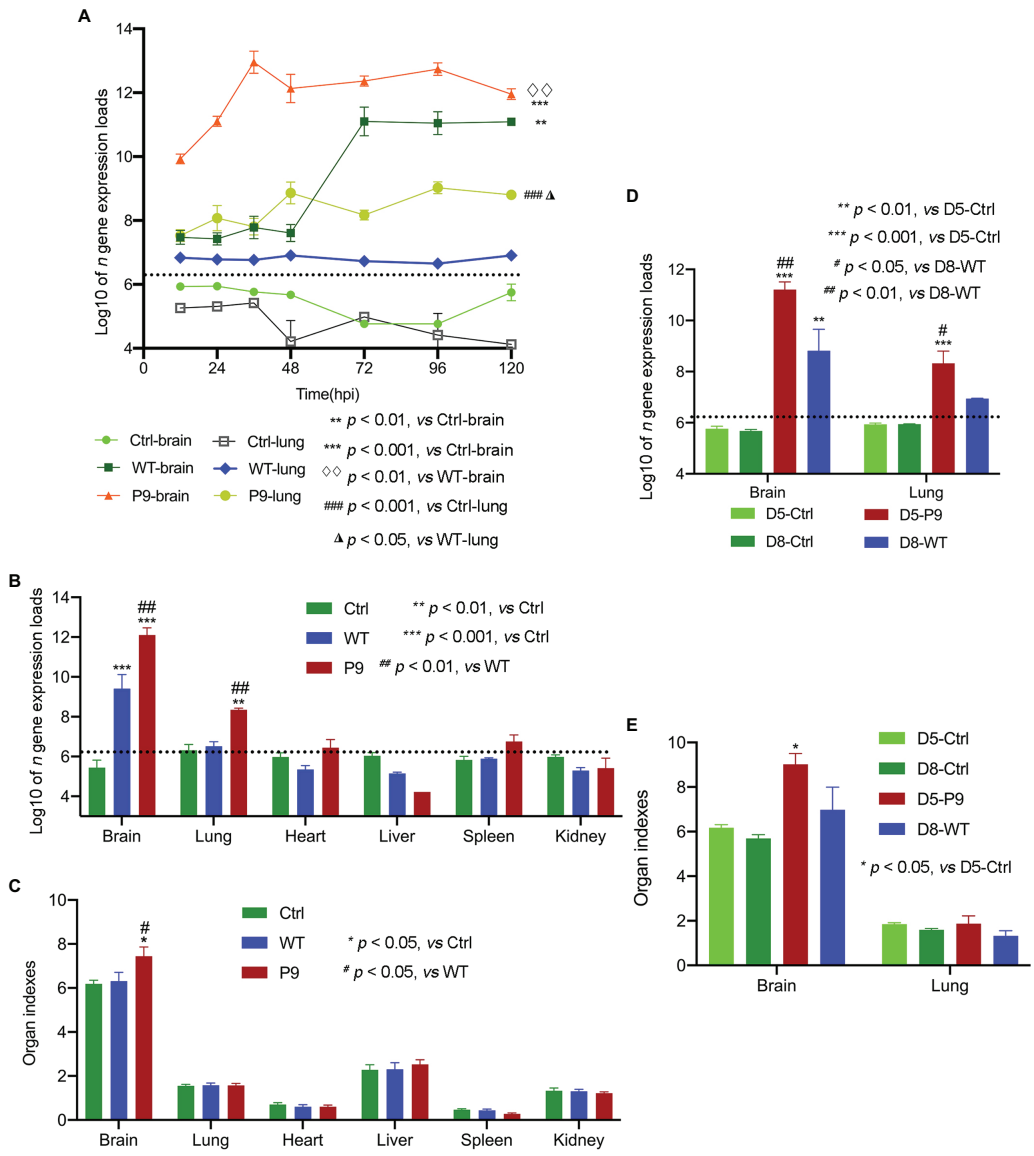
Virus expression and organ indices in various organs of suckling mice. The proliferation kinetics of these viruses in the brain and lungs was analyzed (*n* = 3); **(A)** Virus *n* gene’s copy number **(B)** and organ indices **(C)** in various organs of mice infected with wild-type strain viruses (WT) and adapted-strain viruses (P9) were detected at 4 dpi (*n* = 4), respectively. The viral replication levels **(D)** and organ indices **(E)** in WT-infected suckling mice at 8 dpi and P9-infected at 5 dpi (*n* = 3–4) were calculated. The data for viral *n* gene (copies/g) are presented as log10, mean ± SEM, and dashed lines indicate the detection limit.

The proliferation kinetics of the P9 virus in brain and lung samples were first investigated. The P9 virus multiplied more rapidly and greatly in brain samples than the WT virus (Figure 3A). The highest copy number of *n* gene in P9 group reached 1.73 × 10^13^ copies/g at 36 hpi and then kept at a higher level, whereas the replication level of the WT virus began to significantly increase from 48 hpi but was statistically lower than P9 at all indicated times (Figure 3A, *p* < 0.01). In lung samples, although the expression level of *n* gene of the P9 and WT groups was comparable at 12 hpi, it increased with time in P9 and was considerably higher than that of the WT (*p* < 0.05) and control groups (*p* < 0.001; Figure 3A). The expression of *n* gene in lung samples from WT-infected mice was not significantly higher than those of the normal control group (Figure 3A, *p* > 0.05). Since all mice in P9 group died at 5 dpi, the P9-infected mice were sacrificed and sampled at 4 dpi in subsequent experiments.

To determine the tissue infectivity of the P9 virus, brain, lung, heart, liver, spleen, and kidney samples from P9, WT, and control groups were harvested at 4 dpi to compare the organ indices and viral replication levels. The expression levels of viral *n* gene in the brain and lung samples of the P9 group were significantly higher than those in the WT and control groups (*p* < 0.001 for the brain and *p* < 0.01 for the lung; Figure 3B). However, only the brain index of P9-infected mice was significantly higher than those in both the WT and control groups (Figure 3C, *p* < 0.05). The replication level of WT virus in the brain sample was higher than in the control group (*p* < 0.001), but the difference in the expression of viral *n* gene was not significant in the lung samples (Figure 3B, *p* > 0.05). The copy number of viral *n* gene in other organs of P9- and WT-infected mice was not significantly different from that in the control group (Figure 3B, *p* > 0.05). Since the mean survival time of WT group (7.6 ± 0.163 days) was longer than that of the P9 group (4.5 ± 0.224 days), the organ indices and *n* gene expression levels between WT-infected pups at 8 dpi and P9-infected pups at 5 dpi were compared. The value of these two variables in WT group was still considerably lower than the results in the brain (*p* < 0.01) and lung samples (*p* < 0.05) of the P9 group (Figures 3D,E).

**Figure 4:**
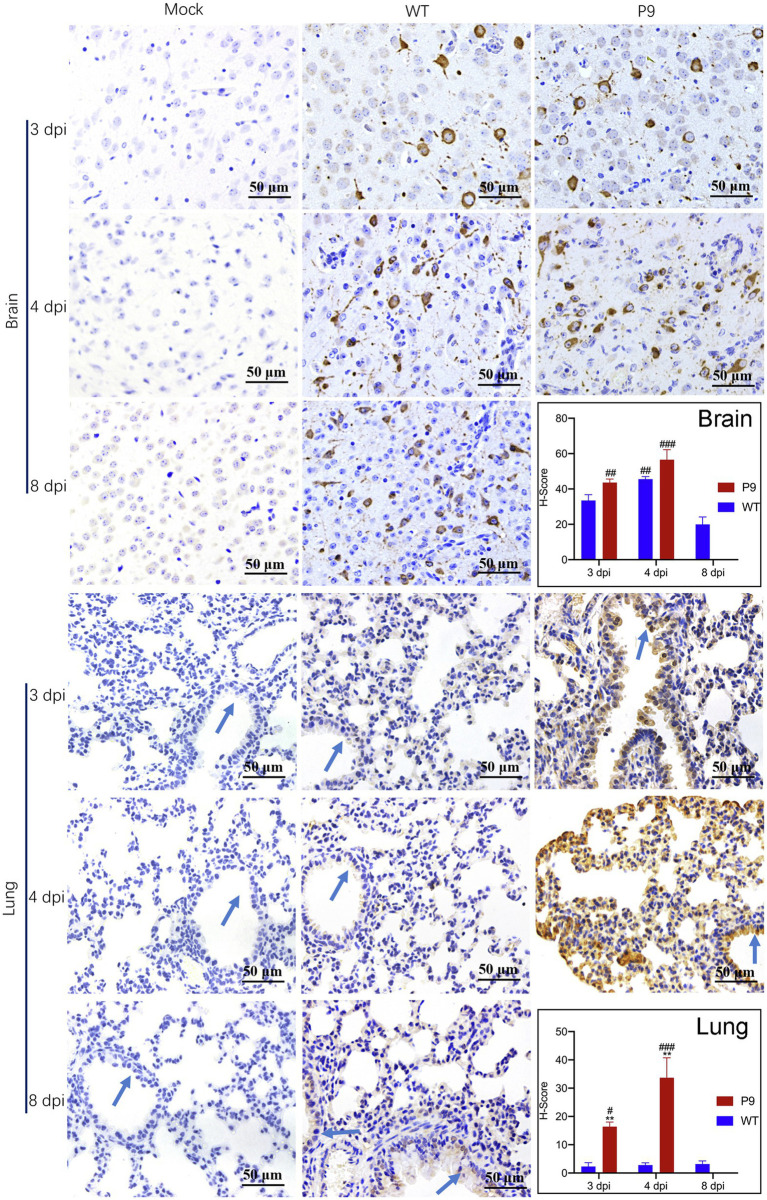
Virus N protein expression and localization in organs of suckling mice. Viral nucleocapsid protein expression and localization in brain (400×) and lung (400×) tissues were examined immunohistochemically with DAB staining at the indicated times (*n* = 3–5). The OC43 N protein expression level was calculated and indicated as H-Scores. Positive expression is shown in tan. Blue arrows, bronchus. ^#^*p* < 0.05, ^##^*p <* 0.01, and ^###^*p* < 0.001, vs. WT-8 dpi group; ^**^p < 0.01, vs. WT-4 dpi group.

Furthermore, we used immunohistochemical analysis to investigate the viral N protein expression in brain and lung tissues with DAB staining. N protein of P9 viruses was abundant in the neurons of the cerebral cortex, and the H-Score was higher than that of WT group at all indicated times (Figure 4). At 3 dpi, weak staining was observed in the tracheal epithelium and a few alveolar septal cells of P9 animals, while the majority of the bronchial and alveolar epithelial cells were distinctly stained at 4 dpi (Figure 4). The expression level of N protein in WT group was relatively lower in lung samples at 3, 4, and 8 dpi (Figure 4). These results indicated that the P9 virus could efficiently replicate in the lungs of suckling mice. When combined with its faster replication rate and higher replication level, the virulence of P9 virus to suckling mice was increased.

**The infection of the P9 virus caused pathological changes and upregulated the production of pro-inflammatory factors in brain and lung tissues.**

**Figure 5:**
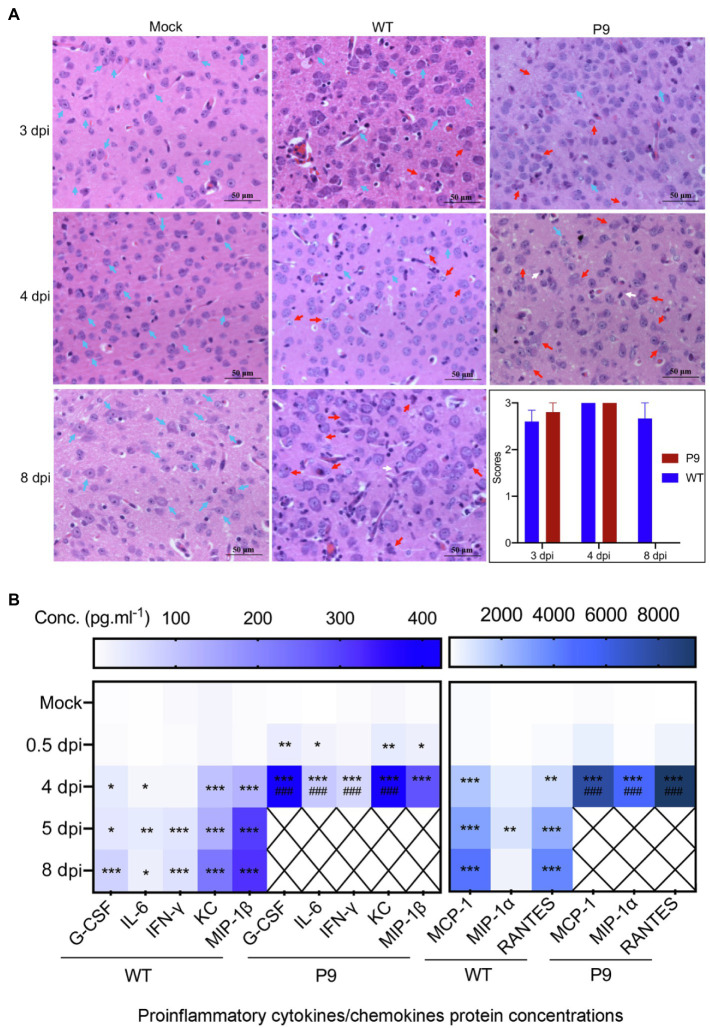
Pathological changes and expression of pro-inflammatory factors in brain tissues induced by virus infection. **(A)** HE staining of brain tissue at 3, 4, and 8 dpi after virus WT or P9 infection, 400×, *n* = 3–5. HE scores were recorded for evaluation of the pathological changes. Blue arrows, normal tissue; red arrows, degenerative neurons; and white arrows, microglia. **(B)** Heat map shows the mean values of expression level of inflammatory cytokines in the brain at 0.5, 4, and 8 dpi after viral infection by the bio-plex assay, mean (*n* = 5). ^*^p < 0.05, ^**^p < 0.01, and ^***^p < 0.001, vs. normal control group (mock); ^###^*p* < 0.001, vs. WT-8 dpi group.

Our findings indicate that the adapted strain (P9) was efficiently replicated in the brain and lungs of suckling mice. Previous research has shown that OC43 infection could cause inflammation in the brain and involve nerve cells in the cerebral cortex (Butler et al., 2006). Therefore, we investigated the pathological and pro-inflammatory factors’ changes in brain and lung tissues. Infection with the P9 virus resulted in inflammatory cell infiltration, a decrease in neuronal cells, and an increase in cell degeneration (Figure 5A). Similar pathological alterations were observed in the brains of WT-infected animals but not in the mock group (normal control group; Figure 5A). The HE scores of brain samples from the P9 group were not statistically different from those of the WT group at 3 and 4 dpi (Figure 5A). At 4 dpi, the expression of cytokines and chemokines G-CSF, IFN-γ, IL-6, KC, MCP-1, MIP-1α, MIP-1β, and RANTES in the brain of suckling mice was significantly upregulated after P9 infection (Figure 5B, *p* < 0.05, or 0.01, 0.001). Increasing expression of inflammatory cytokines was also shown in WT group at 4, 5, and 8 dpi (Figure 5B, *p* < 0.05, or 0.01, 0.001), but the expression level of most cytokines was lower than those in the P9-4 dpi group (Figure 5B, *p* < 0.001). The upregulation degree of all inflammatory factors except MIP-1β in the WT-8 dpi group was lower than that in the P9-4 dpi group (Figure 5B, *p* < 0.001).

**Figure 6:**
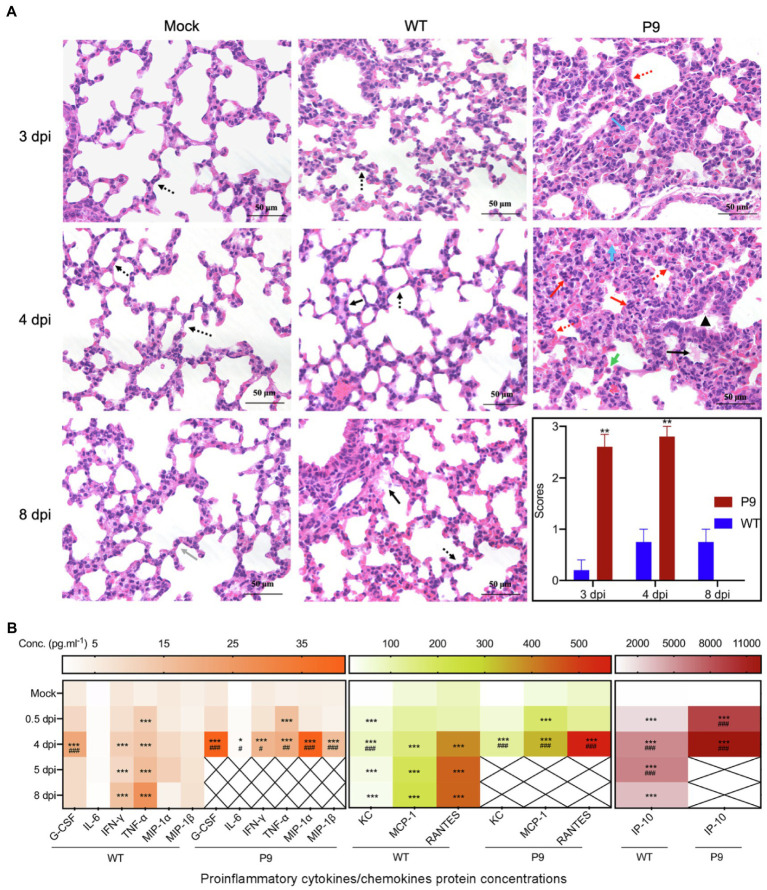
Pathological changes and pro-inflammatory factor expression in lung tissues caused by virus infection. **(A)** HE staining of lung tissue after virus infection at 3, 4, and 8 dpi, 400×, *n* = 3–5. HE scores were recorded for evaluation of the pathological changes. Grey arrows, normal tissue; red arrows, alveolar septal infiltration of inflammatory cells, infiltration of inflammatory cells around the bronchus; yellow arrows, alveolar epithelial hyperplasia and alveolar septum widening; green arrows, suspected virus inclusion body; black arrows, alveolar hyaline membrane; orange arrows, trachea epithelial ulcer; and blue arrows, alveolar interstitial exudation. ^**^p < 0.01, vs. WT-4 dpi group. **(B)** Heat map shows the expression of inflammatory cytokines in the lung at 0.5, 4, and 8 dpi after infection with virus by the bio-plex assay, mean (n = 3–5); ^*^p < 0.05, ^**^p < 0.01, and ^***^p < 0.001, vs. normal control group (mock); ^#^*p* < 0.05, ^##^*p <* 0.01, and ^###^*p* < 0.001, vs. WT-8 dpi group.

In lung samples of P9-infected animals, alveolar septum widening, inflammatory cell infiltration in the alveolar septum, alveolar epithelial hyperplasia, fibrous effusion of the alveolar interstitium, concentration of inflammatory cells near the bronchus, and alveolar hyaline membrane were observed (Figure 6A). These changes were more severe in P9 groups at 3 and 4 dpi, but mild alveolar epithelial hyperplasia and alveolar hyaline plasma membrane were occasionally detected in WT groups at 4 and 8 dpi (Figure 6A, *p* < 0.01). Meanwhile, P9 infection increased the severity of the pulmonary inflammatory cytokine storm, and the chemokines and cytokines of G-CSF, IFN-γ, KC, MCP-1, MIP-1α, MIP-1β, RANTES, IP-10, and TNF-α were significantly and persistently upregulated at 0.5 and 4 dpi (Figure 6B). Following WT infection, G-CSF, IP-10, and KC levels were increased at 4 dpi but decreased at 8 dpi (Figure 6B, *p* < 0.001). In comparison with the control group, IFN-γ, MCP-1, RANTES, and TNF-α were statistically upregulated in WT group (Figure 6B, *p* < 0.001). There was, however, no significant variation in MIP-1α was detected among the three groups, a protein associated with asthma and airway inflammation (Figure 6B, *p* > 0.05; Rojas-Dotor et al., 2013). Except for TNF-α, the other factors in the P9 group at 4 dpi were considerably higher than those of WT at 8 dpi (Figure 6B). These results suggest that P9 virus infection could cause pathological changes and severe cytokine storms in both their brain and lung tissues.

### The Adapted Strain (P9) Had a High Infection Rate in Brain and Lung Samples

To investigate the infection rate of P9, the entire brains and lungs were homogenized with sterile PBS with a weight to volume ratio of 1:10 (g/ml) for qRT-PCR detection of the OC43 *n* gene expression. In 80 mice infected with P9 (1000SMLD_50_ per mouse) viruses, there were 67.5% of pups having CT values of less than 29% and 88.75% of pups with CT values of less than 32 in lung samples, and the *n* gene expression was detected in all brain samples (CT < 29, 100%; Supplementary Figure 1). When CT values of *n* gene were between 32 and 35, N protein expression was hardly detectable by the immunohistochemistry assay, but it was obviously detected when CT values were less than 29. These findings indicate that P9 had a high infection rate under 1,000 SMLD_50_.

### The Adapted OC43 Model Was Useful for Evaluating the Antiviral and Anti-inflammatory Effects of Drugs

**Figure 7:**
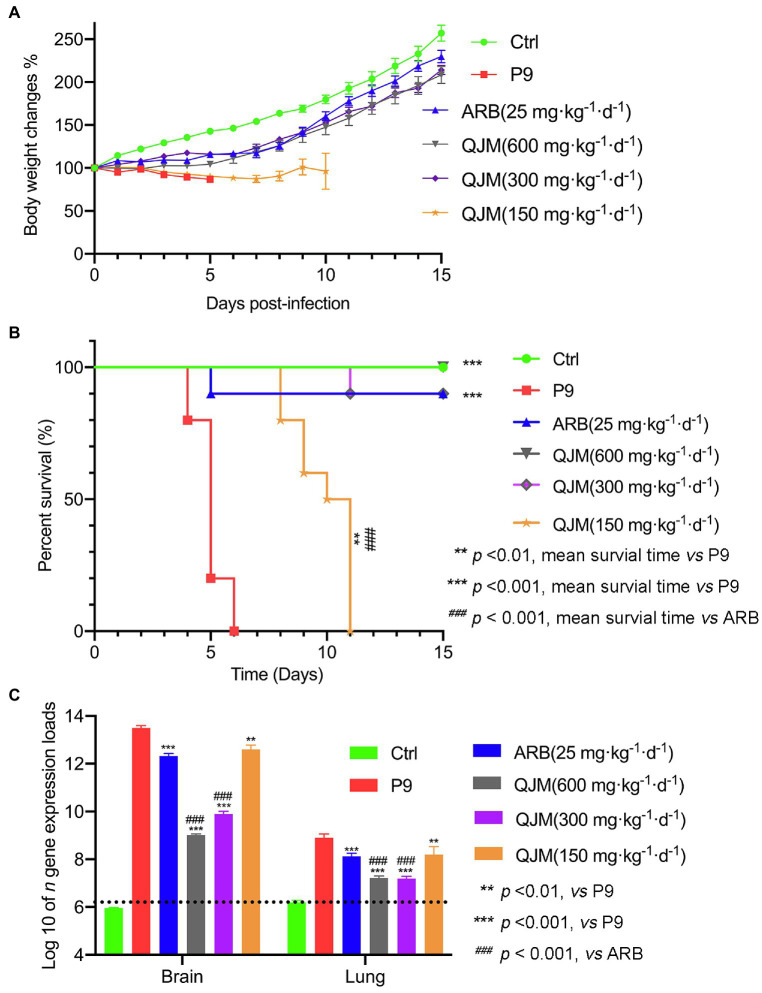
QJM increased the survival rate of P9-infected suckling mice and inhibited the nucleocapsid gene expression. After inoculation with an adapted-strain virus (P9) and treated with ARB and QJM, body weight change **(A)** and survival rate **(B)** of suckling mice within 15 days (*n* = 10) and the copy number of viral *n* gene in the brain and lungs at 4 dpi (**C**; *n* = 3–5). The data for viral *n* gene expression (copies/g) are presented as log10, mean ± SEM, and dashed lines indicate the detection limit.

To explore the application potential of our model for evaluation of antiviral agents, the therapeutic effects of QJM and ARB on pups infected with P9 virus were analyzed. Mice were treated with high (600 mg·kg^−1^·d^−1^), middle (300 mg·kg^−1^·d^−1^), and low (150 mg·kg^−1^·d^−1^) concentrations of QJM and with 25 mg·kg^−1^·d^−1^ of ARB at 2 hpi, respectively. The symptoms, including being reluctant to move and ingest milk, and the swaggering walk of suckling mice that resulted from P9 infection, were improved by drug treatments. The body weight of all infected mice decreased after viral inoculation, but all drug treatments considerably raised the body weight and survival rates to varying degrees (Figures 7A,B). The mice in the ARB group experienced a brief weight loss and subsequently gained weight at 4 dpi. However, one mouse in the ARB group died at 5 dpi, resulting in a 90% (9/10) survival rate (Figures 7A,B). The weight of mice in the high and middle doses of QJM groups began to increase at 5 dpi, and the survival rates were 100% (10/10) and 90% (9/10), whereas the weight of mice in the low-dose group began to rise at 7 dpi, but they still died at 11 dpi, with a mortality rate of 100% (Figures 7A,B). In the low concentration QJM group, the mean survival times were significantly increased when compared with the P9 group (9.9 ± 1.287 days vs. 5.0 ± 0.667, *p* < 0.01; Supplementary Table 4). These findings suggest that both ARB and QJM treatments were able to improve symptoms and the survival rate of P9-infected mice.

**Figure 8:**
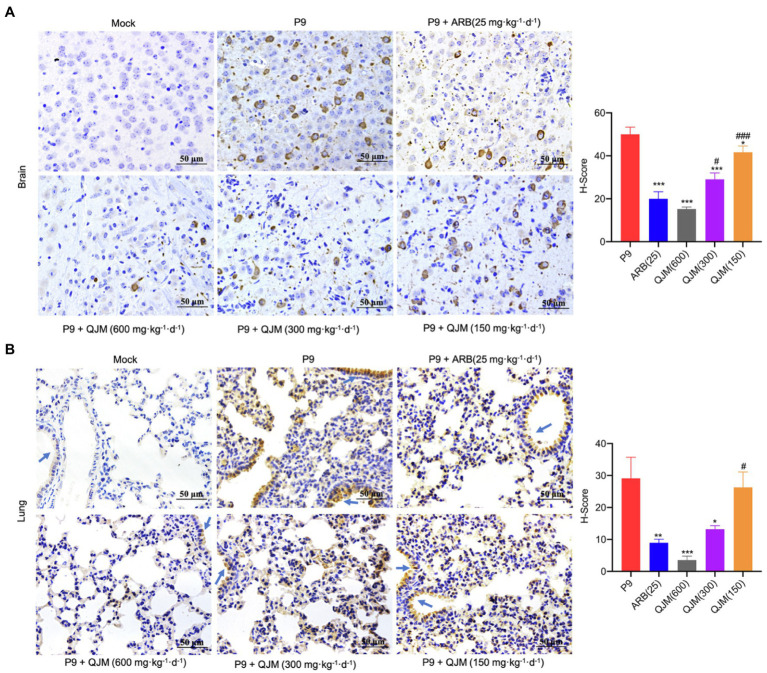
QJM inhibited the N protein expression. N protein expression in brain (400×; **A)** and lung (400×; **B)** tissues (*n* = 3–5) was examined immunohistochemically with DAB staining at 4 dpi. The OC43 N protein expression level was calculated and indicated as H-Scores. Positive expression is shown in tan. Blue arrows, bronchus. ^*^p < 0.05, ^**^p < 0.01, and ^***^p < 0.001, vs. P9 group; ^#^*p* < 0.05 and ^###^*p* < 0.001, vs. ARB group.

**Figure 9:**
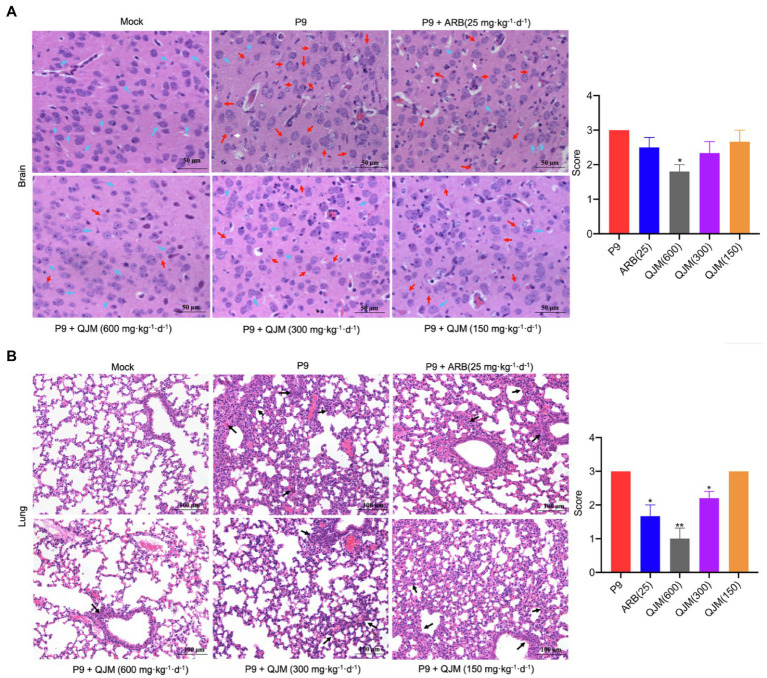
QJM therapy improved the pathological changes in the brain and lungs of P9-infected suckling mice. HE staining shows the pathological changes in brain (400×; **A)** and lung (200×; **B)** tissues at 4 dpi (*n* = 3–5). HE scores were recorded for evaluation of the pathological changes. Blue arrows, normal tissue; red arrows, progressive degeneration of neurons; white arrows, microglia; and black arrows, diseased tissue. ^*^p < 0.05 and ^**^p < 0.01, vs. P9 group.

Furthermore, the effect of ARB and QJM against viral replication in the brain and lung tissues of mice was detected. At 4 dpi, the expression of *n* gene in drug treatment groups was statistically different from those in the P9 group (*p* < 0.01 or 0.001). High and middle concentrations of QJM treatment were more effective in reducing *n* gene expression levels than those of the ARB treatment (*p* < 0.001; Figure 7C). In addition, at 4 dpi, immunohistochemistry assays showed decreased expression of viral N protein in the neurons in the cerebral cortex of the QJM and ARB treatment groups (Figure 8A, *p <* 0.05 or 0.001). The effects of the high dosage of QJM treatment were better than those of the ARB group, but their H-Scores were not statistically different (Figure 8A *p >* 0.5). In lung tissues, N protein was abundantly expressed in the majority of P9-infected bronchial and alveolar epithelial cells, whereas ARB and all QJM treatments significantly lowered the H-Scores (Figure 8B, *p <* 0.05, 0.01, or 0.001). At 4 dpi, inflammatory cell infiltration, severe neuronal cell reduction, and cell degeneration induced by viral infection were significantly reduced by a high dose of QJM treatment (Figure 9A, *p* < 0.05). ARB and the other doses of QJM treatment moderately improved the symptoms, but HE scores were not statistically different from the P9 group (Figure 9A, *p* > 0.05). Meanwhile, high and middle doses of QJM and ARB treatments also significantly reversed pathological changes in lung tissues, including widened alveolar septum, infiltrated inflammatory cells, proliferated alveolar epithelium, fibrous interstitial exudation in the alveolar interstitial space, and inflammatory cells concentrated near the bronchus (Figure 9B, *p* < 0.05 or 0.01).

**Figure 10:**
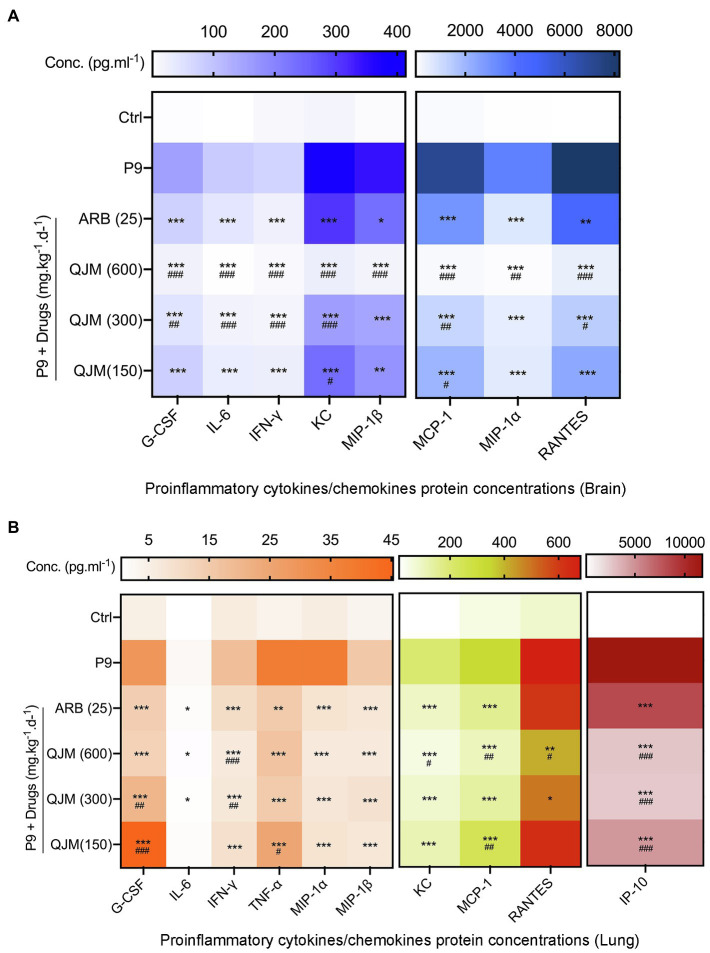
QJM therapy modulated the expression of inflammatory/chemokines in the brain and lungs of P9-infected suckling mice. Heat map: The expression of inflammatory cytokines in the brain **(A)** and lung **(B)** at 4 dpi after treating with ARB and QJM by the bio-plex method, mean (*n* = 3–5); ^*^p < 0.05, ^**^p < 0.01, and ^***^p < 0.001, vs. P9 group; and ^#^*p* < 0.05, ^##^*p* < 0.01, and ^###^*p* < 0.001, vs. ARB group.

QJM and ARB interventions significantly reduced the production of pro-inflammatory cytokines and chemokines at 4 dpi in P9-infected suckling mice. In brain samples, the treatment of QJM and ARB significantly inhibited the production of G-CSF, IFN-γ, IL-6, KC, MCP-1, MIP-1α, MIP-1β, and RANTES (Figure 10A, *p* < 0.05). The high concentration of QJM was more effective than ARB in the suppression of all detected cytokines (Figure 10A, *p* < 0.01 or *p* < 0.001). In lung samples, high and middle dosage QJM treatments significantly reduced the production of G-CSF, IFN-γ, IL-6, IP-10, KC, MCP-1, MIP-1α, MIP-1β, RANTES, and TNF-α induced by P9 infection (Figure 10B, *p* < 0.05, *p* < 0.01, or *p* < 0.001), and the inhibitory role was generally superior to that of ARB (Figure 10B). ARB played a minor role in the production of RANTES (Figure 10B). These results prove the efficacy of QJM and ARB against the HCoV-OC43 infection *in vivo*. Our model was useful when it came to evaluating the antiviral and anti-inflammatory effects of antiviral drugs.

## Discussion

Animal models are critical in antiviral research. In this study, we developed a new mouse-adapted HCoV-OC43 that caused cerebral and pulmonary diseases in suckling C57BL/6 mice with a reduced survival period. By sequence comparison with the WT virus, 15 nucleic acid mutations resulting in 8 coding changes were detected in P9 virus, and mutations were concentrated in the S protein. The S protein of HCoVs contains significant viral neutralisation epitopes and the interaction between this protein and its binding receptor determines the host tropism and pathogenicity of CoVs (Woo et al., 2009; Song et al., 2020). For β-CoVs, the S protein is split into S1 and S2 subunits by furin-like proteases at the conserved cleavage site of RRSRR/G. The degree of sequence variation in S1 is extremely high, whereas S2 sequences are conserved. Our result was in accordance with a previous study that 8 nucleic acid mutations in the S region of the P9 virus were all located in the S1 subunit. There are four domains in the S1 subunit, with domains A and B in OC43 serving as receptor binding domains (Peng et al., 2012; Tortorici et al., 2019). Domain A is also referred to as the N-terminal domain, which forms a complex with the glycogroup 5-N-acetyl-9-O-acetylneuraminic acid on the cell surface that allows virus invasion (Peng et al., 2012). Among 5 coding changes resulting from 8 nucleic acid mutations in P9 virus, 3 coding mutations (Y250H, N259D, and D264K) are located inside domain A, and a recent study indicated that numerous mutations in domain A may have a synergic effect on virus virulence (Brison et al., 2011). Another 2 mutations (A394G and K494I) were located within domain B, which was reported to have the highest variability across CoVs and correlates to the ability of different viruses to interact with distinct host receptors (Tortorici et al., 2019). Further, mutations in domain B were associated with changes in viral tropism and influenced animal-to-human transmission of SARS (Qu et al., 2005; Hulswit et al., 2016). Another 2 amino acid changes were in NSP3 and NSP6 within the ORF1a region. NSP3 is a multi-domain transmembrane protein and is responsible for cleavage of pp1ab *via* papain-like protease (PL^Pro^) and block host innate immune response (Serrano et al., 2009). It has been reported that the synonymous mutation in NSP3 (F106F) could affect the fitness of the virus (Tomaszewski et al., 2020). NSP6 decreases the autophagic capacity of infected cells, which provides an innate defense against viral infections (Mercatelli and Giorgi, 2020). Most recently, the accumulation of mutations in S1 subunit of S protein was found to be associated with ORF1a: 3,675–3,677 deletion and this deletion was indicated as an adaptive mutation that facilitates the process of SARS-CoV-2 evolution (Kistler et al., 2022). One coding mutation was found in N protein. The *n* gene mutation was positively correlated with the severity of coronavirus cases and a high number of deaths seen in South American countries like Brazil (Burki, 2020; Vahed et al., 2021). Besides, the mutations R203K and G204R in the N protein contributed to the improved viral fitness of the COVID-19 B.1.298 variant (Plante et al., 2021). Since viral infection is a game between the virus and the host cells, the influence of the identified coding mutations on expanded tropism and enhanced virulence of P9 viruses should be verified in future work.

An ideal animal model for infectious diseases should exhibit similarities to humans in terms of symptoms, infection routes, and the link between viral replication level and disease severity, as well as practical advantages such as low cost, a clear genetic background, and ease of manipulation (Gretebeck and Subbarao, 2015). The laboratory mouse is a useful and affordable model that has been developed for HCoV-OC43 infection since the distribution of 9-O-acetylated sialic acid receptors, the attachment receptor for this virus, in mice is most similar to that of humans (Wasik et al., 2017; Lang et al., 2020). However, as one of the most common respiratory pathogens, the viral replication level in pulmonary tissues and the occurrence of lung inflammation and respiratory illness in reported HCoV-OC43 mouse models were not determined (Jacomy and Talbot, 2001, 2003; Jacomy et al., 2006). In present study, the infected mice presented lethargy and stooped posture, which were similar to some symptoms of human beings. Besides cerebral infectivity, the pulmonary invasiveness of P9 virus was demonstrated by qRT-PCR detection of viral replication in lung tissues, and the expression of the viral *n* gene increased with time. Histopathological and immunohistochemical examinations confirmed the occurrence of pneumonia. In addition, compared with WT infection, the P9 virus resulted in lower body weight and shorter survival time, as well as a higher viral multiplication level in brain samples. The copy numbers of viral *n* gene reached 3.96 × 10^13^ copies/g and 1.03 × 10^11^ copies/g in brain and lung samples, respectively. This viral replication level was comparable with 2 reported fatal cases associated with this virus, and the viral loads were 3.49 × 10^6^ to 1.10 × 10^10^ copies/ml in the respiratory tract specimens of a 75-year-old patient with fatal pneumonia, and Ct values ranged from 22 to 24 in an immunocompromised child with fatal encephalitis (Nilsson et al., 2020; Lau et al., 2021). It should be noted that the infection was conducted by intracerebral inoculation, which was different from the natural route of infection. We tried intranasal inoculation and found that it was easy to cause asphyxia in suckling mice, whereas intracerebral inoculation of suckling mice benefited the success rate of modeling. Although the infection method did not mimic natural infection way, our adapted OC43 model developed encephalitis and pneumonia and exhibited similar tissue tropism, symptoms, and inflammation response to humans. Compared to reported mouse models, this model provides a useful tool for pathogenesis studies of respiratory and brain diseases caused by HCoV infection.

Besides the association of the severity and mortality of HCoV infection with a high viral replication level, the pathological role of the immune response triggered by infection has been increasingly recognized (Zhang et al., 2020). Tissue injury and inflammatory alterations are required phenotypes for lung disease in animal models (Wong et al., 2004; Channappanavar and Perlman, 2017; Mahallawi et al., 2018). In this study, P9 infection led to the occurrence of typical viral pneumonia symptoms (Figure 4). It also resulted in a large decrease in neuronal cells, which was consistent with previous studies (Jacomy and Talbot, 2003; Butler et al., 2006). Moreover, it stimulated the expression of inflammatory cytokine storms in brain and lung samples, such as G-CSF, IFN-γ, KC, MCP-1, MIP-1α, MIP-1β, RANTES, IP-10, and TNF-α, which were similar to the inflammation response induced by HCoV-infected patients (Wong et al., 2004; Channappanavar and Perlman, 2017; Mahallawi et al., 2018). A previous study found that the S1 subunit of SARS-CoV-2 interacted with the human ACE2 receptor to activate the NF-κB pathway, which upregulated the expression of pro-inflammatory cytokines and chemokines (IL-1β, TNF-α, IL-6, and MCP-1) and resulted in epithelial damage in human bronchial epithelial cells (Hsu et al., 2020a). Over-expression of a range of pro-inflammatory cytokines and chemokines would cause inflammatory cell activation and infiltration, increase vascular permeability, and, eventually, lead to pulmonary edema and pneumonia (Kircheis et al., 2020). For instance, during the acute inflammatory response to a virus infection, the body releases the cytokines TNF-α, IL-1, and IL-6, which act on fibroblasts and endothelial cells, hence increasing vascular permeability (Gulati et al., 2016; Kawano et al., 2017). Further, the expression of MIP-1α and MCP-1 by macrophages, T cells, monocytes, and other inflammatory cells enhanced the release of IL-6 or IL-8, which contributed to the pathogenic process of bronchiectasis (Barnes, 2008; Rui Jin and Miao, 2017; Yibing Niu, 2018). IP-10 is thought to be a biomarker for the severity of COVID-19 (Chen et al., 2020). Following virus infection, alveolar macrophages and neutrophils can emit a substantial amount of IP-10 to attract T cells that express the IP-10 receptor CXCR3 to the infected region, accelerating the onset, and progression of lung tissue inflammation (Liangdi Hu et al., 2019). Meanwhile, inflammatory factors expressed in the brain, such as IL-6 and IFN-γ in the cerebral white matter, can activate inflammatory responses, leading to neurodegeneration and the onset of multiple sclerosis (Gulati et al., 2016). These findings may help to explain the degenerative changes in nerve cells and the development of neuropathic gait in suckling mice following P9 infection. Based on previous studies, our results suggested that P9 infection partially mimicked the clinical symptoms, viral replication, and pathological features observed in HCoV patients. Therefore, this model is valuable for elucidating the mechanisms of tissue damage and inflammation induced by viral infection.

Mice infected with adapted viruses have been proven to be accessible and reproducible for evaluating the efficacy and safety of antiviral therapies for viral infections (Day et al., 2009; Dinnon et al., 2020). The death rates, body weight loss, viral replication level, production of pro-inflammatory factors, and pathogenic alterations in the brain and lung tissues of P9-infected pups might be useful indicators for antiviral and anti-inflammatory drug screening. We employed the model to look into the pharmacology of QJM and ARB *in vivo*. Previous study found that QJM and ARB showed a strong antiviral and anti-inflammatory effect of OC43 *in vitro* (Xie et al., 2021). In this study, QJM and ARB treatment raised the survival rates and suppressed the expression of OC43 nucleocapsid protein genes and their proteins, while decreasing G-CSF, IFN-γ, IP-10, KC, MCP-1, MIP-1α, RANTES, and TNF-α. In combination of our previous study, we proved that QJM and ARB have both *in vivo* and *in vitro* antiviral and anti-inflammatory effects against HCoV-OC43 infection. This model was useful for assessing the efficacy of antiviral drugs.

This study established a novel mouse-adapted OC43 model that is practical and repeatable for antiviral research and successfully used it to evaluate the efficacy of QJM, which demonstrated this TCM had antiviral and anti-inflammatory activities against OC43 *in vivo*. In addition, the amino acid mutations discovered in ORF1ab, ORFS, and ORFN may be required for the adapted virus to become more virulent. Future research may investigate whether these mutations are linked to the extended lung tissue tropism. Furthermore, because of the difference in immune responses between suckling mice and adult mice, our model is suitable for drug research at a young age stage. Our model provides a choice for future anti-coronavirus drug screening and vaccine evaluation.

## Data Availability Statement

The datasets presented in this study can be found in online repositories. The names of the repository/repositories and accession number(s) can be found in the article/Supplementary Material.

## Author Contributions

XX and SD conceived and designed the study, are responsible for study coordination, and obtained funding and ethical approval. PX and YF developed the study design, revised the protocol, and wrote the original draft. ZB revised the manuscript. PX, YF, HY, ZZ, RL, and TZ conducted the experiment. RL performed the data curation, review, and editing the manuscript. ZY and JZ obtained the resources. All authors contributed to the article and approved the submitted version.

## Funding

This work was supported by National Key Research and Development Program of China (2022YFC0867400), National Natural Science Foundation of China (82041005), the Key Research and Development Program of Yunnan Province (202003 AC100006), and Major Science and Technology Projects of Yunnan Province (2019ZF004).

## Conflict of Interest

The authors declare that the research was conducted in the absence of any commercial or financial relationships that could be construed as a potential conflict of interest.

## Publisher’s Note

All claims expressed in this article are solely those of the authors and do not necessarily represent those of their affiliated organizations, or those of the publisher, the editors and the reviewers. Any product that may be evaluated in this article, or claim that may be made by its manufacturer, is not guaranteed or endorsed by the publisher.

## Supplementary Material

The Supplementary Material for this article can be found online at: https://www.frontiersin.org/articles/10.3389/fmicb.2022.845269/full#supplementary-material. The GenBank accession numbers for genome sequences of WT and P9 viruses discussed in this paper are HCoV-OC43 WT sequence (ON376724) and HCoV-OC43 P9 sequence (ON376725).

Supplementary Figure 1 The infection rate of P9 virus in brains and lung samples. The copy number of OC43 *n* gene was detected by qRT-PCR at 4 dpi in brain and lung samples of suckling mice infected with P9 virus (*n* = 80).

Supplementary Figure 2 Graphical abstract of this study.

## Abbreviations

WT: wild-type virus
P9: adapted strain virus
ORF: open reading frame
NSP: non-structural protein, Spike gene
He: Hemagglutinin-esterase gene
n: nucleocapsid gene
N protein: Nucleocapsid protin
ARB: Arbidol
QJM: Qingwenjiere Mixture
TCM: traditional Chinese medicine
Ctrl: normal control group
SMLD_50_: the median lethal dose of suckling mice
HE: hematoxylin and eosin
DAB: 3,3’-Diaminobenzidine
hpi: hours post-infection
dpi: days post-infection
S protein: Spike glycoprotein
TCID_50_: 50% tissue culture infective dose
