# Supplementary figures and images for: A Mouse-Adapted Model of HCoV-OC43 and Its Usage to the Evaluation of Antiviral Drugs
Source: Front Microbiol. 2022 May 17;13:845269. doi: 10.3389/fmicb.2022.845269 (PMC9220093; doi:10.3389/fmicb.2022.845269)

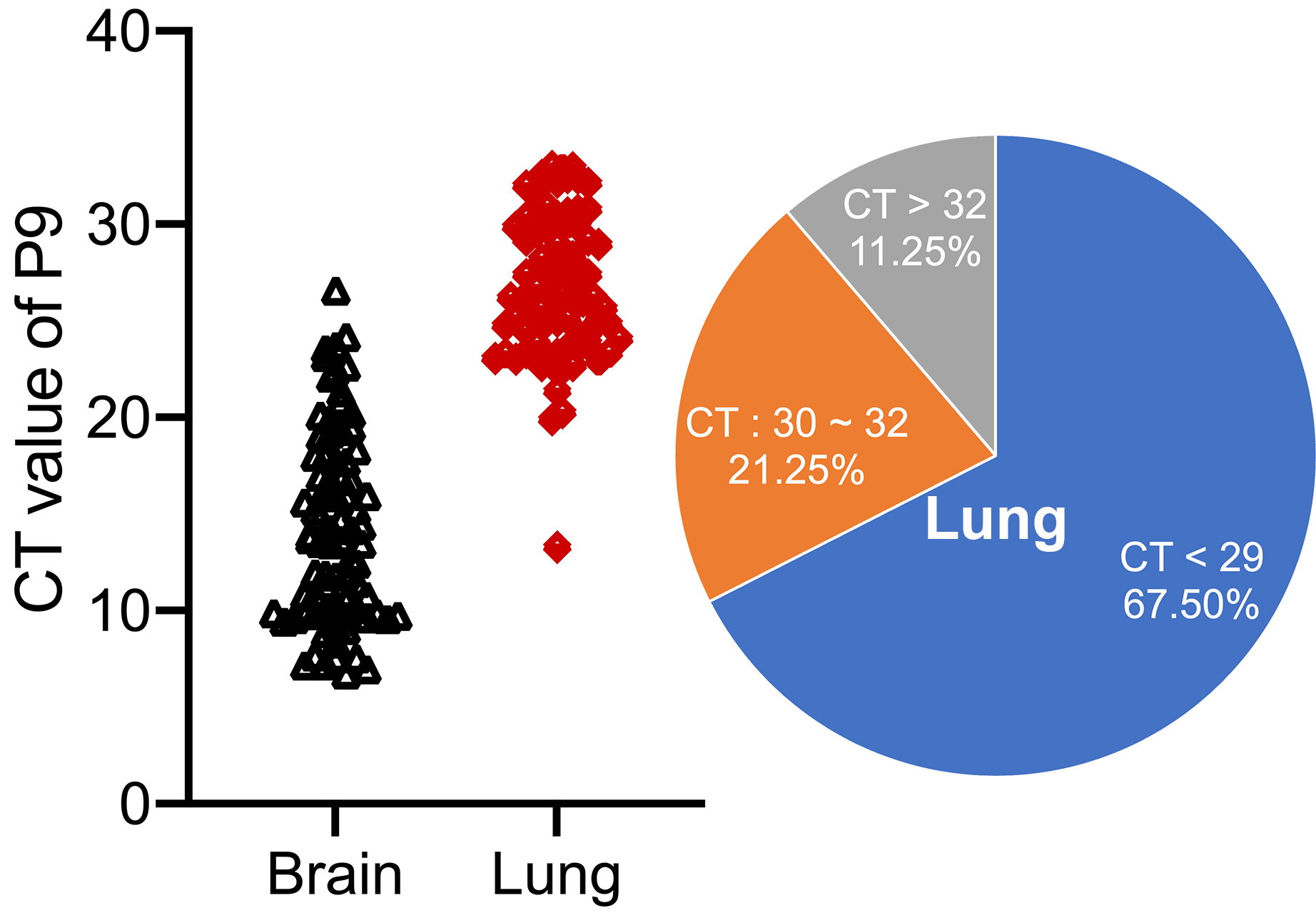

Supplement: Supplementary Figure 1 — The infection rate of P9 virus in brains and lung samples. The copy number of OC43 n gene was detected by qRT-PCR at 4 dpi in brain and lung samples of suckling mice infected with P9 virus (n = 80). [file Data_Sheet_1.ZIP › Supplementary Figure 1. Infection rate of P9.jpg]

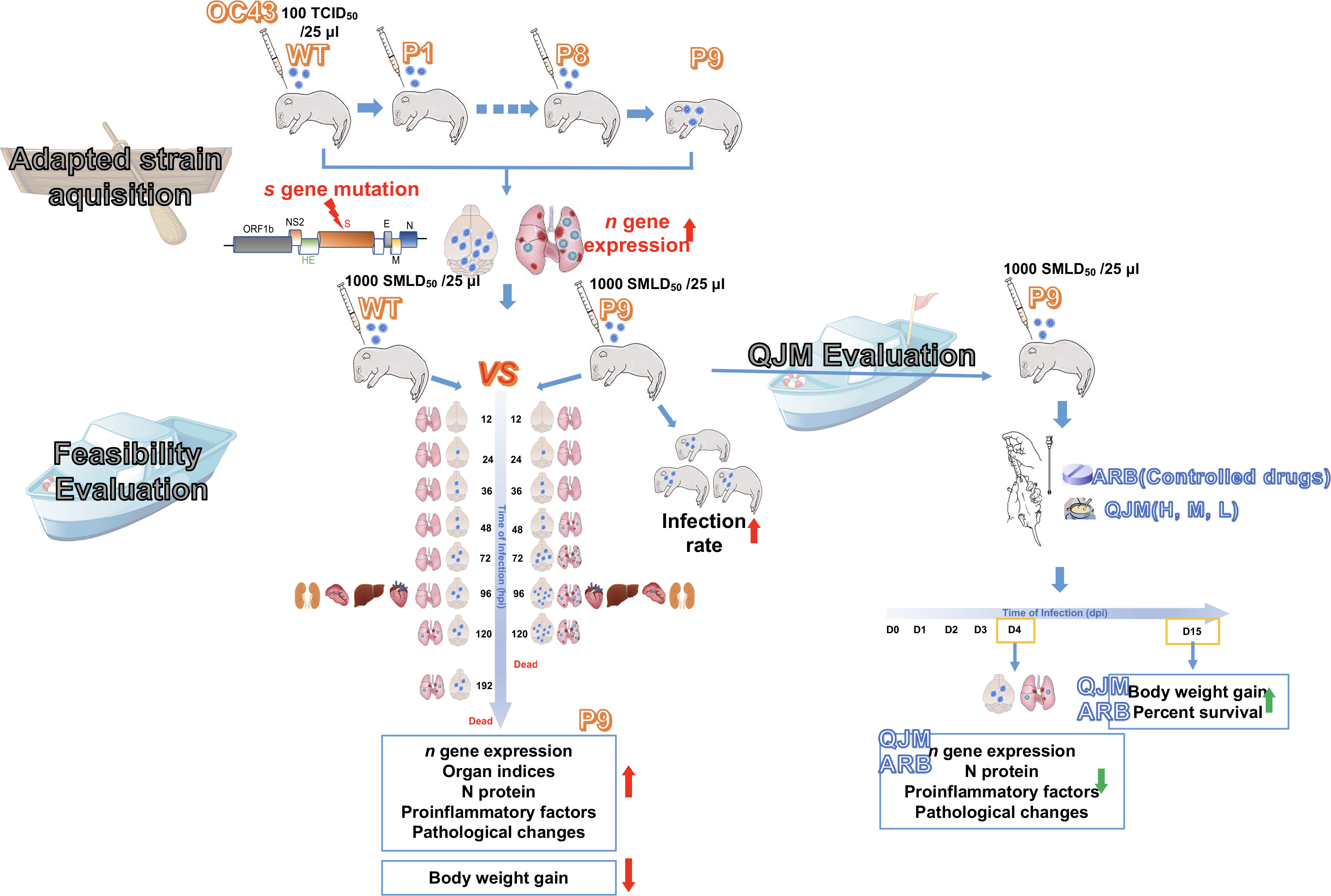

Supplement: Supplementary Figure 1 — The infection rate of P9 virus in brains and lung samples. The copy number of OC43 n gene was detected by qRT-PCR at 4 dpi in brain and lung samples of suckling mice infected with P9 virus (n = 80). [file Data_Sheet_1.ZIP › Supplementary Figure 2. Graphical abstract.jpg]
