# Supplementary material for: A Mouse-Adapted Model of HCoV-OC43 and Its Usage to the Evaluation of Antiviral Drugs
Source: Front Microbiol. 2022 May 17;13:845269. doi: 10.3389/fmicb.2022.845269 (PMC9220093; doi:10.3389/fmicb.2022.845269)
Supplement: Supplementary Figure 1 — The infection rate of P9 virus in brains and lung samples. The copy number of OC43 n gene was detected by qRT-PCR at 4 dpi in brain and lung samples of suckling mice infected with P9 virus (n = 80). [file Data_Sheet_1.ZIP › Supplementary Tables-2022-4-16-11-22.doc]

**Supplementary Table 1 The primer sequences for HCoVs genes using in this study**

| Target | Primer | Sequences (5’-3’) | Length (bp) |
| --- | --- | --- | --- |
| Hemagglutinin-esterase and partial S1 subunit | OC43-HE-Forward | TCATTTCCGTAAGGCTTGTCA | 2652 |
|  | OC43-HE-Reverse | ATGACTGCAAATAGCCCAAATT |  |
| Partial S1 and S2 subunit | OC43-S_1- Forward | TGTTTATTTAGGCATGGCRCT | 2201 |
|  | OC43-S_1- Reverse | ARGTGGCAGCCAAAGTRTAT |  |
| Partial S1 and S2 subunit | OC43-S_2- Forward | TCACAGTTGGTTGAATATGGT | 1806 |
|  | OC43-S_2- Reverse | TACGGCACCAAGCATGACTA |  |
| HCoV-OC43,  Nucleocapsid | Forward | ATGTTAGGCCGATAATTGAGGACTAT | 68 |
|  | Reverse | AATGTAAAGATGGCCGCGTATT |  |
|  | Taqman probe | FAM- CATACTCTGACGGTCACA AT-NFQ-MGB | - |
| Whole genome sequence | OC-1F | CATCCACTCCCTGTAATCTAT | Variable |
|  | OC-1R | AACTGATAGGCCACATGCTTA |  |
|  | OC-2F | ATGCTATKTGAGAGGTTGTAATCC |  |
|  | OC-2R | TATGCAATACAGGRTTAACAGG |  |
|  | OC-3F | GGTTGGATTCCTGGTAACATGA |  |
|  | OC-3R | TTTTCTGCCTGARAGACAAATTC |  |
|  | OC-4F | TTGCACAAGCATTTCAGAGTGTT |  |
|  | OC-4R | GCACATACAGACCCAAAACTTTAAC |  |
|  | OC-5F | GATGTAGAAATGTCGGATTTTG |  |
|  | OC-5R | TAAAGACACATCAGAAGGCA |  |
|  | OC-6F | AGAACACAGGGTAAACAAAGTT |  |
|  | OC-6R | CAAGATCYTCACGACTCAATGW |  |
|  | OC-7F | TTGCATGTAAATGTGGTGTAA |  |
|  | OC-7R | ATCAAAACCCAACTTAGMATTA |  |
|  | OC-8F | SGAGAAAGTAGATGGTGTGTAT |  |
|  | OC-8R | CGCACAGCTTTAACCACATT |  |
|  | OC-9F | TGATGTGTATGATATGTGGCTTA |  |
|  | OC-9R | TAATCCACTGTGCAATCTTA |  |
|  | OC-10F | TGAGCAGAGCAGTTAACGTACCTA |  |
|  | OC-10R | TGGATAAAACCATGCYGTATACA |  |
|  | OC-11F | GAAGCTGATAGGAGAGCATT |  |
|  | OC-11R | AAATATGTTGGCACCAAGTTA |  |
|  | OC-12F | CGTAAAAGTTKTTCTATTGATTCAG |  |
|  | OC-12R | TCTGCTTATTATAAGTAAGCTTCAG |  |
|  | OC-13F | TATATGGTCTGTGGATGCTTT |  |
|  | OC-13R | AAGCACACAGCCACTAGCAT |  |
|  | OC-14F | CCCTACCAAAGTGTTACGATA |  |
|  | OC-14R | CACACCACACAATCACRTTA |  |
|  | OC-15F | CTGGTGCTATACTYGCTGTAA |  |
|  | OC-15R | AAGCWGTAGGHGGTTGGTMA |  |
|  | OC-16F | GCTTGCTCTCAGTTGGCTAA |  |
|  | OC-16R | CCATTGAAGTCAGTACCAGTAT |  |
|  | OC-17F | AAACCACAAGGAGCYTTTCATGTAA |  |
|  | OC-17R | CTACCCATAATCTGACGTCCTTGG |  |
|  | OC-18F | CAATGGATTTAGCCAAGTTA |  |
|  | OC-18R | ACAGCAACCCACTTAGCAAT |  |
|  | OC-19F | GGTACAAGGGTTCTAACTTAGAGG |  |
|  | OC-19R | TAAGCAGATTTAGCAATATTACAGG |  |
|  | OC-20F | CGTTGAATATGARGTTGCTAA |  |
|  | OC-20R | CMGCATAGGTAACATAGAYRTT |  |
|  | OC-21F | TGTGTACCATTGAATGCAATA |  |
|  | OC-21R | GACCYTTAGCATCTTGAACAG |  |
|  | OC-22F | CTTAGTGATGTTGATGGTCTTA |  |
|  | OC-22R | AACTTCCATCCCGCCAAAAT |  |
|  | OC-23F | AGTTTGTACARGTGCCTGTAGGT |  |
|  | OC-23R | TCCACAWAAATTTGCCTRACAAG |  |
|  | OC-24F | GGAGTATGCCATATCATCCTAACAC |  |
|  | OC-24R | CAATRCGTAGTARGTTTGGCATAGC |  |
|  | OC-25F | CTGGGATGATATGTTACGCCG |  |
|  | OC-25R | GTGTGTTGTGAACARAAYTCRTG |  |
|  | OC-26F | TAAGTGCCTTTCAACAGGT |  |
|  | OC-26R | KGCCTTTTGCGTTTCTGC |  |
|  | OC-27F | ACAGGATCTCCGTACATAGAYGA |  |
|  | OC-27R | TTTCATAAACCAAGGCGGAC |  |
|  | OC-28F | GCACCACGTGTGTTATTGAG |  |
|  | OC-28R | TTAAAYTGTTCACCAGGAGGA |  |
|  | OC-29F | GTGCYCATGCCACGCKTGATA |  |
|  | OC-29R | TAGCATCCATRCCATCCATATACACACA |  |
|  | OC-30F | CACCKAATGCAGTTGTATGTAGAT |  |
|  | OC-30R | TAACACGGCTTTGTGTAAAGATAG |  |
|  | OC-31F | GTGCGTAAAGAAGGTSAGGATGT |  |
|  | OC-31R | GAATAGTACCARCAGGCAACC |  |
|  | OC-32F | GAAGCCAGTTACTTTGCCTACAG |  |
|  | OC-32R | TTTATAGTCTTCAGGCTGTATRTCC |  |
|  | OC-33F | ATGCAGACAAGCCTAATCATTTTAT |  |
|  | OC-33R | CGGAAATGAAAACTTTTCCTGTAG |  |
|  | OC-34F | AGCTAGAAGAAGGYGATCTTCC |  |
|  | OC-34R | TRAGACCATAWATAACACCAGTGTC |  |
|  | OC-35F | CCTGCTTATATAGCTCCTCAAGC |  |
|  | OC-35R | CATAATAAGTACCCAAACCATTA |  |
|  | OC-36F | CCYRTTTGTRTGYATGAYCC |  |
|  | OC-36R | YGCATAAAARGTACCACC |  |
|  | OC-37F | CTRCTATARYTATAGGTAGT |  |
|  | OC-37R | GCCCAAATTRCCCAATTGTAGG |  |
|  | OC-38F | TGATGCTGCTAAGATATATGG |  |
|  | OC-38R | ATTYCGARATAGCAATGCTGG |  |
|  | OC-39F | GTTGAGGTTAATGCGACTTATTAT |  |
|  | OC-39R | GACACCAAGMCCATTAAT |  |
|  | OC-40F | GACCTCATTTGTGTGCAAAG |  |
|  | OC-40R | GAAGGTTGGRTRTTGARGTG |  |
|  | OC-41F | ATACTTGGATGTACACTGGTAGTG |  |
|  | OC-41R | ATRTAACGGAGAWATTTCTTCTCA |  |
|  | OC-42F | TCCAATCTAGCATTTGTTACCACG |  |
|  | OC-42R | AACAGCAAAACCACTAGTATCGCTT |  |
|  | OC-43F | ACTAGGTAYTGGCTATTCTTTGGC |  |
|  | OC-43R | GCATCCTTGCCAAGTTTTGC |  |
|  | OC-44F | GGGTTACTATATTGAAGGCTCAGG |  |

**Supplementary Table 2 Mutations identified in Spike-cDNA from WT and seral passages of viruses**

| Generations | Nucleotide changes | | | | | | | | |
| --- | --- | --- | --- | --- | --- | --- | --- | --- | --- |
|  | 748 | 775 | 790 | 792 | 1181 | 1481 | 1713 | 1966 | 1998 |
| WT | T | A | G | T | C | A | A | A | C |
| P1 | T | A | G | T | C | A | A | A | C |
| P2 | C | A | G | T | C | A | A | C | C |
| P3 | C | A/G | A/G | T/G | C/G | A | A | C | C |
| P4 | C | A/G | A/G | T/G | C/G | A | C | A | T |
| P5 | C | A/G | A/G | T/G | C/G | A/T | C | A | T |
| P6 | C | A/G | A/G | T/G | C/G | A/T | C | A | C |
| P7 | C | A/G | A/G | T/G | C/G | T | C | A | T |
| P8 | C | G | A | G | G | T | C | A | T |
| P9 | C | G | A | G | G | T | C | A | T |
| Amino acid changes | Y250H | N259D | D264K | D264K | A394G | K494I | NM | NM | NM |

NM, nonsense mutation

**Supplementary** Table 3. Lethal effects of adapted strain P9 on Suckling mice

| Group | SMLD_50_ | Survival/Total mice | Mean survival time *^a^*±SD (day) |
| --- | --- | --- | --- |
| Normal Control | 0 | 10/10 | >15 |
| WT | 1000 | 0/10 | 7.60±0.163 |
| P9 | 1000 | 0/10 | 4.5±0.224^***^ |

^a^Compared to the WT group, ^***^ *p* <0.001

**Supplementary** Table 4. The protective effect of drugs against

infected with OC43 adapted strain P9 in suckling mice

| Drugs | SMLD50 | Survival/Total quantity | Mean survial time *^b^* ±SD (day) |
| --- | --- | --- | --- |
| Normal Control | 0 | 10/10 | 15.0±0.000 |
| P9 | 1000 | 0/10 | 5.0±0.667 |
| ARB  (25 mg·kg^-1^·d^-1^)  ARB | 1000 | 9/10 | 14.0±3.162*** |
| QJM  (600 mg·kg^-1^·d^-1^) | 1000 | 10/10 | 15.0±0.000*** |
| QJM  (300 mg·kg^-1^·d^-1^) | 1000 | 9/10 | 14.6±1.265*** |
| QJM  (150 mg·kg^-1^·d^-1^) | 1000 | 0/10 | 9.9±1.287** |

^a^ Compared to the P9 group, ** *p* <0.01, *** *p* <0.001

^b^ The average amount of days that mice survived after infection within 15 days
